# Supplementary material for: Comprehensive Annotation and Functional Exploration of MicroRNAs in Lettuce
Source: Front Plant Sci. 2021 Dec 24;12:781836. doi: 10.3389/fpls.2021.781836 (PMC8739914; doi:10.3389/fpls.2021.781836)
Supplement: Supplementary file 2 [file Data_Sheet_8.ZIP › GSM2230749.plot/Lsa-miRN21_Lsat_1_v5_gn_8_24801.1_2427_TPlot.pdf]

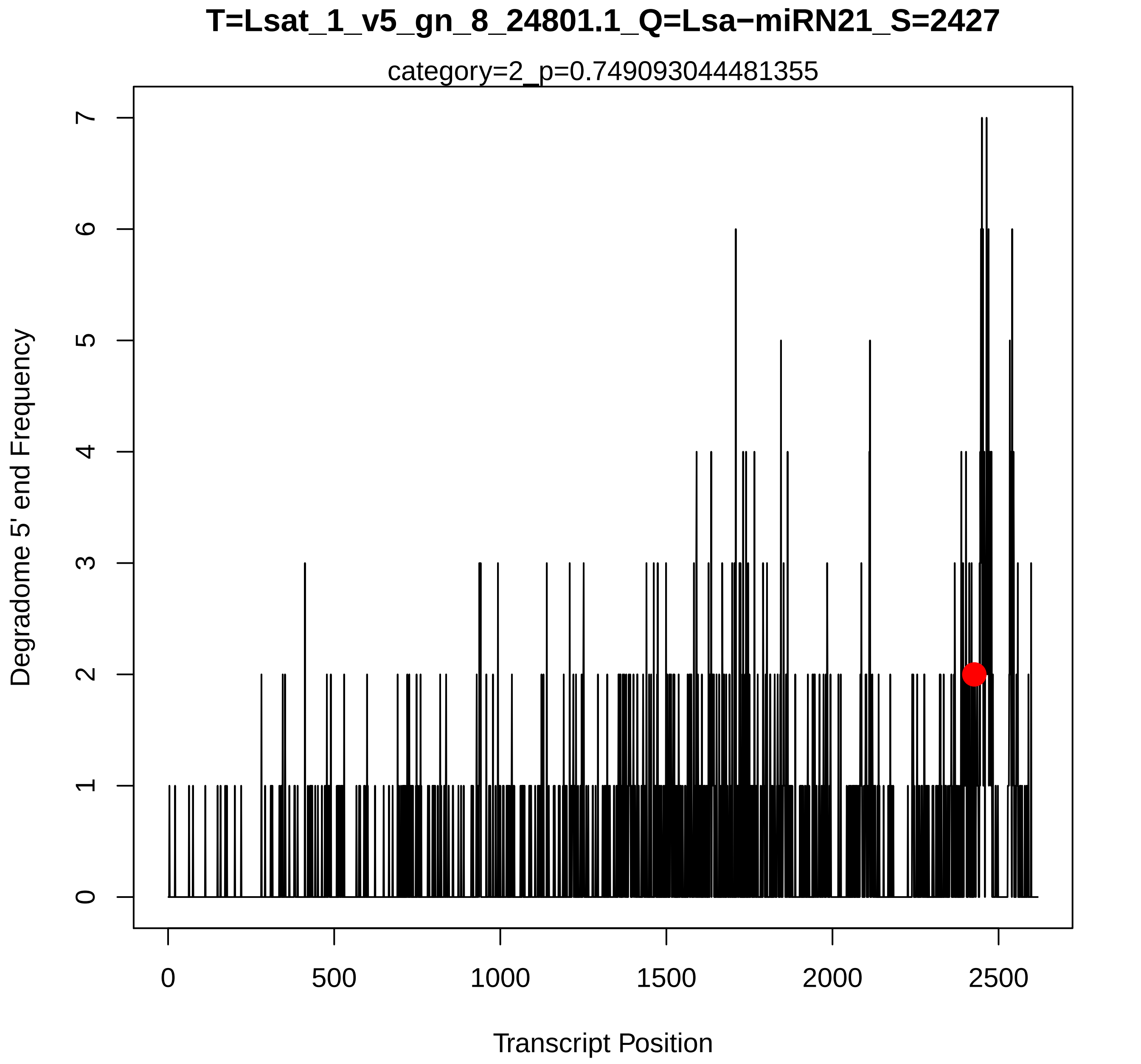

T=Lsat_1_v5_gn_8_24801.1_Q=Lsa−miRN21_S=2427
category=2_p=0.749093044481355
Degradome 5' end Frequency
0
1
2
3
4
5
6
7
0
500
1000
1500
2000
2500
Transcript Position
